# Precise localization value of lumbar lamina and ligamentum flavum boundaries in minimally invasive tubular resection of intraspinal schwannomas

**DOI:** 10.3389/fneur.2025.1721714

**Published:** 2026-01-26

**Authors:** Longfei Shu, Yan Liu, Feihu Dai, Chunmei Chen, Yuhai Wang, Wei Zhao

**Affiliations:** 1 Department of Neurosurgery, Wuxi Clinical College of Anhui Medical University, 904th Hospital of Joint Logistic Support Force of PLA, Wuxi, China; 2 Department of Orthopedics, Jingjiang Chinese Medicine Hospital, Taizhou, China; 3 Department of Neurosurgery, The Second Affiliated Hospital of Fujian University of Traditional Chinese Medicine, Fuzhou, China

**Keywords:** lamina, ligamentum flavum, lumbar vertebrae, minimally invasive spinal surgery, schwannoma, tubular approach

## Abstract

**Objective:**

This study aimed to assess the utility of lumbar lamina and ligamentum flavum boundaries as anatomical landmarks for the precise localization and resection of lumbar intraspinal schwannomas using a minimally invasive tubular approach.

**Methods:**

We conducted a retrospective analysis of 17 patients who underwent surgical resection between September 2021 and September 2023. Preoperative imaging was used to determine the optimal lamina landmarks relative to the tumor’s poles or midpoint. The boundaries or specific sites of the ligamentum flavum subsequently guided the precise drilling of the bone window. We recorded intraoperative parameters, including retractor inclination angle, operative time, and blood loss. Patient outcomes were assessed during a two-year follow-up using the Oswestry Disability Index (ODI), MRI to evaluate resection, and X-ray to assess spinal stability.

**Results:**

All tumors were successfully resected without neurological complications. The mean operative time was 119.7 ± 14.7 min, mean blood loss was 47.1 ± 11.9 mL, and the mean retractor angle was 6.3 ± 2.5°. After a mean follow-up of 30.9 ± 1.6 months, ODI scores showed significant improvement, decreasing from 31.5 ± 5.4% to 14.9 ± 3.4%. Postoperative MRI confirmed gross-total resection in all cases, and X-rays revealed no spinal instability.

**Conclusion:**

The boundaries of the lumbar lamina and ligamentum flavum are reliable and effective anatomical landmarks. Utilizing these landmarks facilitates precise, minimally invasive resection and is correlated with favorable short-term outcomes.

## 1 Introduction

The posterior minimally invasive approach utilizing non-expandable tubular retractors with an inner diameter of 1.6–1.8 cm is a well-established technique for treating lumbar degenerative diseases (1–5). However, its use for resecting lumbar intraspinal schwannomas is less common (6–9), owing to several technical challenges. First, unlike degenerative pathologies, schwannomas lack a fixed position within the spinal canal and vary considerably in size. This variability prevents surgeons from relying on standard surgical trajectories for tumor exposure. While intraoperative fluoroscopy can guide initial tubular retractor placement over the lamina, the subsequent microscopic view is often restricted to a single lamina. This limited view makes it difficult to determine the precise extent of bone that must be removed to adequately expose the tumor. Second, the skin incision is not always reliable. In patients with thick subcutaneous fat or elderly individuals with loose skin, sterile drapes can traction the skin, displacing the planned incision from its preoperatively targeted site. Without intraoperative correction, this can lead to inadequate surgical exposure.

Consequently, identifying specific, reliable anatomical landmarks is essential for intraoperative trajectory correction and precise tumor localization. The superior and inferior edges of the lumbar lamina and the boundaries of the ligamentum flavum represent critical anatomical junctions (10–15). These structures are easily identifiable within the spinal canal and offer real-time spatial orientation under the microscope. We hypothesize that these landmarks would provide reliable guidance. Our technique involves analyzing preoperative imaging to determine the spatial relationship between the tumor’s poles (or midpoint) and the nearest superior or inferior edge of the lamina. This analysis identifies the optimal bony landmark within the initial tubular field. Subsequently, the spatial relationships between the tumor poles and the nearest boundary or specific anatomical point of the ligamentum flavum, guide the precise extent of laminectomy performed with a high-speed drill, enabling accurate tumor localization and complete resection. This retrospective case series analyzes our initial experience with this technique to evaluate its feasibility and outcomes.

## 2 Materials and methods

### 2.1 Participants

This retrospective study analyzed the clinical and radiological data of patients who underwent minimally invasive tubular resection for lumbar intraspinal schwannomas. The cohort included patients who underwent surgery between September 2021 and September 2023 and completed a minimum two-year follow-up. The study protocol received approval from the Institutional Ethics Committee of our hospital (Approval No.: 20231015). All procedures adhered to the ethical principles of the Declaration of Helsinki. The inclusion criteria were: (1) a single, well-circumscribed intradural lesion at the lumbar level; (2) homogeneous enhancement of the tumor margins on contrast-enhanced MRI; (3) a tumor sagittal diameter of <3 cm. The exclusion criteria were: (1) MRI findings suggestive of tumor adhesion to the dura mater or a dural tail sign; (2) a tumor sagittal diameter of ≥3 cm; (3) a spinal dumbbell-shaped tumor configuration.

### 2.2 Anatomical landmarks selection and radiological evaluation

**Figure 1:**
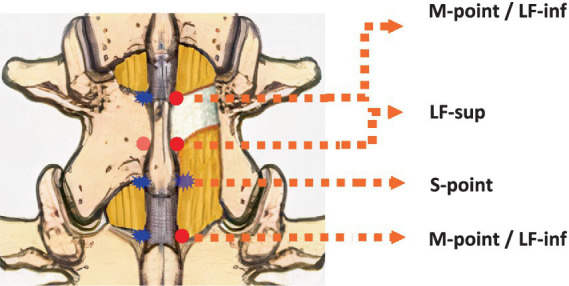
Schematic of anatomical landmarks of the lamina and ligamentum flavum boundaries in the posterior view of a single lumbar spinal segment. M-point/LF-inf, junction of the spinous process base and the superior edge of the lamina, which also corresponds to the inferior attachment site of the ligamentum flavum from the superior adjacent segment. LF-sup, midline superior boundary of the ligamentum flavum. S-point, junction of the spinous process base and inferior edge of the lamina.

We defined a single lumbar spinal segment as the region extending from the superior edge of one vertebral lamina to the superior edge of the lamina of the vertebra directly below it. To facilitate precise topographic description and intraoperative localization, we defined two key anatomical reference points. The M-point: The junction of the spinous process base and the superior edge of the lamina. This point also corresponds to the inferior attachment site of the ligamentum flavum from the superior adjacent segment. The S-point: The junction of the spinous process base and the inferior edge of the lamina, which serves as a key anatomical reference point for the ligamentum flavum. Following laminectomy, exposure of the interlaminar space and the entire ligamentum flavum reveals the anatomical architecture of the posterior spinal canal within a single segment (Figure 1).

**Figure 2:**
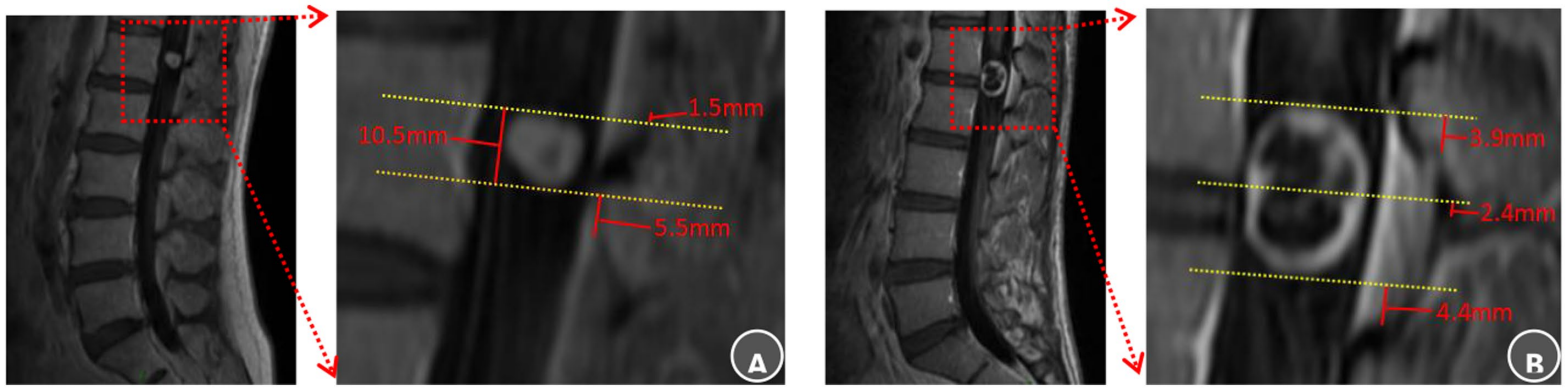
Anatomical landmarks of the lamina and ligamentum flavum boundaries selection and radiological evaluation. **(A)** L1 intraspinal schwannoma: The superior tumor pole was closest to the superior margin of the L1 lamina, recorded as T_s_ = L1-sup – 1.5 mm, It was also 1.5 mm inferior to the T12 ligamentum flavum (T_s_ = T12 LF-inf − 1.5 mm). The inferior pole lay 5.5 mm superior to the L1 ligamentum flavum inferior border (T_i_ = L1 LF-inf + 5.5 mm). The surgical approach was planned using the superior L1 lamina as the cranial guidance. Laminectomy commenced superiority to expose the upper pole and proceeded caudally within two-thirds of the channel diameter to reveal the inferior pole, ensuring preservation of the L1 ligamentum flavum. **(B)** L1–2 intraspinal schwannoma: The tumor midportion was closest to the L1 lamina inferior margin (T_m_ = L1-inf + 2.4 mm). The superior pole was 3.9 mm superior to the L1 ligamentum flavum (T_s_ = L1 LF-sup + 3.9 mm), while the inferior pole was 4.4 mm superior to the L2 ligamentum flavum inferior border (T_i_ = L2 LF-inf + 4.4 mm). Surgical planning designated the inferior L1 lamina as the midpoint reference. Bone removal began at this point, continued until exposing the L1 ligamentum flavum superior boundary with an additional 5 mm resection, and extended caudally only to the L1 ligamentum flavum inferior border.

Two senior attending surgeons independently performed all radiological evaluations using the CareStream PACS system (Centricity, GE Healthcare). Preoperative magnetic resonance imaging (MRI) was analyzed to identify the optimal bony landmark for surgical planning. This involved determining the spatial relationships between the tumor’s superior pole, inferior pole, or geometric midpoint and the nearest superior or inferior edge of the lamina. The measurement protocol on sagittal MRI was as follows: Lines were drawn parallel to the adjacent vertebral endplates to establish a consistent plane of reference. The vertical distance from each tumor reference point (superior pole, inferior pole, or midpoint) to the selected bony landmark was measured. A positive value denoted a location superior to the bony reference; a negative value denoted an inferior location. This same convention was applied to measure the spatial relationships between the tumor poles and the nearest boundary of the ligamentum flavum or the predefined S-point. Each surgeon performed all measurements in duplicate, and the mean values were used for the final analysis (Figure 2).

### 2.3 Surgical incision design

**Figure 3:**
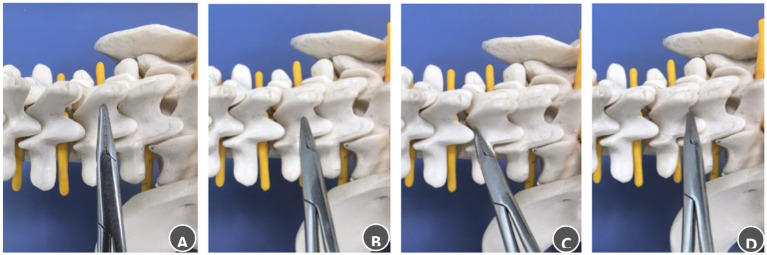
Steps for palpating bony landmarks in the posterior lumbar spinal canal. **(A)** Advance the clamp tip medially along the lumbar spinous process to locate the midline. **(B)** Slide the clamp tip caudally along the spinous process to the spinous process–lamina junction. **(C)** Move the clamp tip cephalad along the junction between the spinous process root and the lamina, a distinct loss of resistance indicates the M-point. **(D)** Move the clamp tip caudally along the same junction; a distinct loss of resistance indicates the S-point.

The initial skin incision was planned using lateral fluoroscopy with a C-arm. A longitudinal incision (1.8–2.0 cm in length) was marked 1.5 cm lateral to the midline on the side of the tumor mass, corresponding to the surface projection of the vertebral body or disc space adjacent to the tumor. A 6–8 mm initial skin incision was made at the center of this line. Through this incision, a clamp was introduced and directed medially to palpate the spinous process. The instrument tip was advanced inferiorly to the spinous process–lamina junction. It was then moved cephalad to locate the M-point (characterized by a subtle loss of resistance at the superior lamina edge) or caudally to identify the S-point (a similar sensation at the inferior edge) (Figure 3). The incision was then fully extended according to the spatial relationship between the identified landmark and the tumor. If the landmark corresponded to the tumor midpoint, the incision was extended symmetrically. If it aligned with a tumor pole, the landmark was positioned at the corresponding cranial or caudal end of the incision. The tubular retractor was then inserted along the final incision to ensure the tumor was centered within the operative field.

### 2.4 Surgical procedure

**Figure 4:**
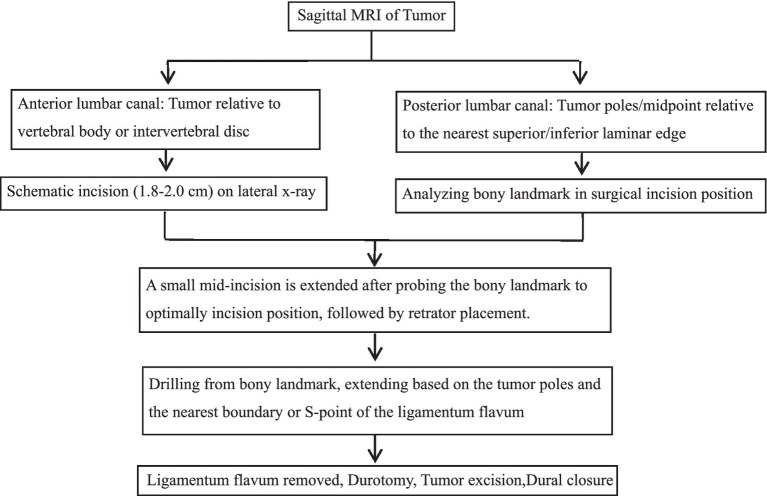
Surgical flowchart: Precision localization via laminar and ligamentum flavum boundaries for percutaneous minimally invasive resection of lumbar spinal schwannoma.

All procedures were performed under general endotracheal anesthesia with the patient in a prone position. The operating table was adjusted to achieve a horizontal alignment of the target vertebral body. Intraoperative neurophysiological monitoring (IONM) was employed throughout. Incision placement and tubular retractor insertion were performed as previously described, with confirmation of the surgical level via lateral fluoroscopy. Laminectomy was initiated from the predefined bony landmark (M- or S-point) using a high-speed drill. The extent of bone removal was guided by preoperative MRI-based measurements between the tumor poles and the ligamentum flavum boundaries or S-point. Following removal of the ligamentum flavum and epidural fat, a midline dural incision was made with careful release of cerebrospinal fluid. The durotomy was extended to expose the tumor poles, and the retractor was repositioned to center the tumor. The final retractor angle was recorded fluoroscopically. Microsurgical tumor dissection was performed with IONM guidance. The tumor and involved nerve root were resected en bloc. Watertight dural closure was achieved with 7–0 Prolene sutures (Ethicon, Inc.), reinforced with an artificial dural graft and fibrin glue. Wound closure was performed in layers. Postoperative care included straight leg raising exercises beginning on postoperative day 1, assisted ambulation with a brace at 3–4 days, and discharge by 6–7 days postoperatively (Figure 4).

### 2.5 Clinical and radiological evaluation

Perioperative data, including operative time, blood loss, and significant IONM changes were recorded. Localization accuracy was assessed by the retractor’s sagittal inclination angle on fluoroscopy after optimal tumor exposure and graded as excellent (<10°), good (10–20°), fair (20–30°), or poor (≥30°). Clinical outcomes were evaluated using ODI scores preoperatively and at 2 years. Follow-ups at 3 months and 2 years included dynamic lumbar radiographs for stability and contrast-enhanced MRI to assess resection and recurrence.

### 2.6 Statistical analysis

Statistical analysis was performed using SPSS software (version 19.0; IBM Corp.). Continuous variables that were normally or approximately normally distributed were presented as mean ± standard deviation. Comparisons between pre- and postoperative groups were conducted using the Student’s *t*-test. A *p*-value of less than 0.05 was considered statistically significant.

## 3 Results

### 3.1 Demographics and characteristics

This study included 17 patients, comprising 9 males and 8 females, with a mean age of 54.2 ± 17.5 years (range: 20–83). Tumor locations were distributed as follows: six at L1, three at L1–2, one at L2, three at L2–3, two at L3, one at L4, and one at L4–5. The most frequent clinical manifestations included low back pain in 15 patients, radicular leg pain in 10 patients, and perineal numbness in 4 patients.

### 3.2 Clinical and radiographic outcomes

The tumors had a sagittal diameter ranging from 7.8 to 28.7 mm. The M-point was used as a cranial reference in 4 cases and a midpoint in 2; the S-point served as the midpoint in 7 cases and caudal reference in 3. One case used the midpoint between M and S. The mean operative time was 119.7 ± 14.7 min, with a mean blood loss of 47.1 ± 11.9 mL and a mean retractor angle of 6.3 ± 2.5°. No neurological injuries, cerebrospinal fluid (CSF) leakage, or infections occurred (Tables 1, 2).

**Table 1:** Details of the landmarks and tumor relations in lumbar intraspinal schwannomas.

Case no.	Sex	Age (yr)	Seg	Tumor size (mm) Sag × Tran × AP	Optimal lamina landmark (mm)	T_s_-FL landmark distance (mm)	Ti-FL landmark distance (mm)	Incision planning
1	F	82	L1	17.2 × 10.2 × 6.5	T_s_ = L1-sup + 2.1	T12 LF-inf + 2.1	L1 LF-sup + 3.1	L1 M-point as cranial incision guide
2	M	52	L1	17.8 × 10.5 × 11.5	T_m_ = L1-sup + 1.2	T12 LF-S − 1.5	L1 LF-sup + 5.2	L1 M-point as midpoint incision guide
3	M	52	L1	10.5 × 8.5 × 7.1	T_s_ = L1-sup − 1.5	T12 LF-inf − 1.5	L1 LF-sup + 5.5	L1 M-point as cranial incision guide
4	M	33	L1	20.1 × 13.7 × 12.5	T_s_ = L1-sup + 2.1	T12 LF-inf + 2.1	L1 LF-S + 0	L1 midpoint as incision midpoint guide
5	F	61	L1	7.8 × 10.1 × 8	T_i_ = L1-inf + 3.6	L1 LF-sup + 5.6	L1 LF-S + 3.6	L1 S-point as caudal incision guide
6	M	64	L1	10.2 × 6.5 × 4.6	T_m_ = L1-sup + 1.5	T12 LF-S + 0	L1 LF-sup + 3.5	L1 M-point as midpoint incision guide
7	F	83	L1-2	24.5 × 11.5 × 10.4	T_m_ = L1-inf + 2.0	L1 LF-sup + 6.4	L1 LF-inf + 2.8	L1 S-point as midpoint incision guide
8	M	42	L1-2	19.1 × 14 × 12	T_m_ = L1-inf + 2.4	L1 LF-sup + 3.9	L1 LF-inf + 4.4	L1 S-point as midpoint incision guide
9	M	48	L1-2	28.7 × 15.3 × 12.5	T_m_ = L1-inf + 2.6	T12 LF-inf + 0	L1 LF-inf + 2.5	L1 S-point as midpoint incision guide
10	F	61	L2	6.8 × 6.1 × 6.5	T_s_ = L2-sup + 1.8	L1 LF-inf + 1.8	L1 LF-inf − 5.0	L2 M-point as cranial incision guide
11	M	20	L2-3	27.8 × 14.5 × 13.5	T_m_ = L2-inf + 3.1	L1 LF-inf + 0	L2 LF-inf + 6.6	L2 S-point as midpoint incision guide
12	M	35	L2-3	17.9 × 13.5 × 11.5	T_m_ = L2-inf − 2.3	L2 LF-sup + 0	L2 LF-S − 7.9	L2 S-point as midpoint incision guide
13	F	82	L2-3	14.9 × 11.2 × 8.4	T_m_ = L2-inf + 1.5	L2 LF-sup + 0	L2 LF-S − 5.4	L2 S-point as midpoint incision guide
14	F	53	L3	15.2 × 12 × 11.2	T_s_ = L3-sup + 4.0	L2 LF-inf + 4.0	L3 LF-sup + 6.0	L3 M-point as cranial incision guide
15	F	56	L3	8.4 × 6.5 × 9.6	T_i_ = L3-inf + 1.0	L3 LF-sup + 4.5	L3 LF-S + 1.0	L3 S-point as caudal incision guide
16	F	54	L4	19.4 × 14.2 × 9.1	T_m_ = L4-sup + 1.0	L3 LF-S + 4.4	L4 LF-sup + 2.4	L4 S-point as midpoint incision guide
17	M	43	L4-5	18.1 × 10.1 × 8.4	T_i_ = L4-inf − 1.0	L4 LF-sup + 7.1	L4 LF-S − 1.0	L4 S-point as caudal incision guide

Sag, sagittal size; Tra, transverse size; AP, anterior–posterior size; T_s_, the superior pole of the tumor; T_i_, the inferior pole of the tumor; T_m_, the midpoint of the tumor; L(n)-sup, the superior edge of the lamina (n); L(n)-inf, the inferior edge of the lamina (n); LF-sup, the superior boundary of the ligamentum flavum; LF-inf, the inferior boundary of the ligamentum flavum; LF-S, the S-point of the ligamentum flavum.

**Table 2:** Lumbar intraspinal schwannoma surgical data and clinical outcomes.

Segment	Case number	Retractor inclination angle (°)	Operative time (min)	Blood loss (mL)	Preoperative ODI (%)	Final follow-up ODI (%)
L1	6	6.3 ± 3.2	110.8 ± 13.2	38.3 ± 11.3	32.7 ± 5.6	14.3 ± 3.4^*^
L1-2	3	3.5 ± 1.2	133.3 ± 12.6	53.3 ± 7.6	30.3 ± 7.8	15.0 ± 4.0^*^
L2 + L2-3	4	7.2 ± 1.1	122.5 ± 17.1	52.5 ± 11.9	32.3 ± 6.2	15.3 ± 3.8^*^
L3-5	4	7.7 ± 1.4	120.1 ± 10.8	50.2 ± 10.8	32.3 ± 4.4	15.6 ± 4.2^*^
Total	17	6.3 ± 2.5	119.7 ± 14.7	47.1 ± 11.9	31.5 ± 5.4	14.9 ± 3.4^*^

Values are presented as mean ± standard deviation. ODI, Oswestry Disability Index.

* *p* < 0.05, statistically significance differences.

### 3.3 Comparison of patient outcomes

All 17 patients were followed for a mean of 30.9 ± 1.6 months (range: 24–48). Low back pain resolved within 1 week in all 15 affected patients, and radicular leg pain improved within 3 months in all 10 cases. Perineal numbness resolved in three of the four patients, with one reporting persistent symptoms. The mean ODI significantly decreased from 31.5 ± 5.4% preoperatively to 14.9 ± 3.4% (*p* < 0.01). MRI confirmed complete resection in all cases without recurrence, and radiographs showed no spondylolisthesis or deformity (Table 2).

### 3.4 Case presentation

**Figure 5:**
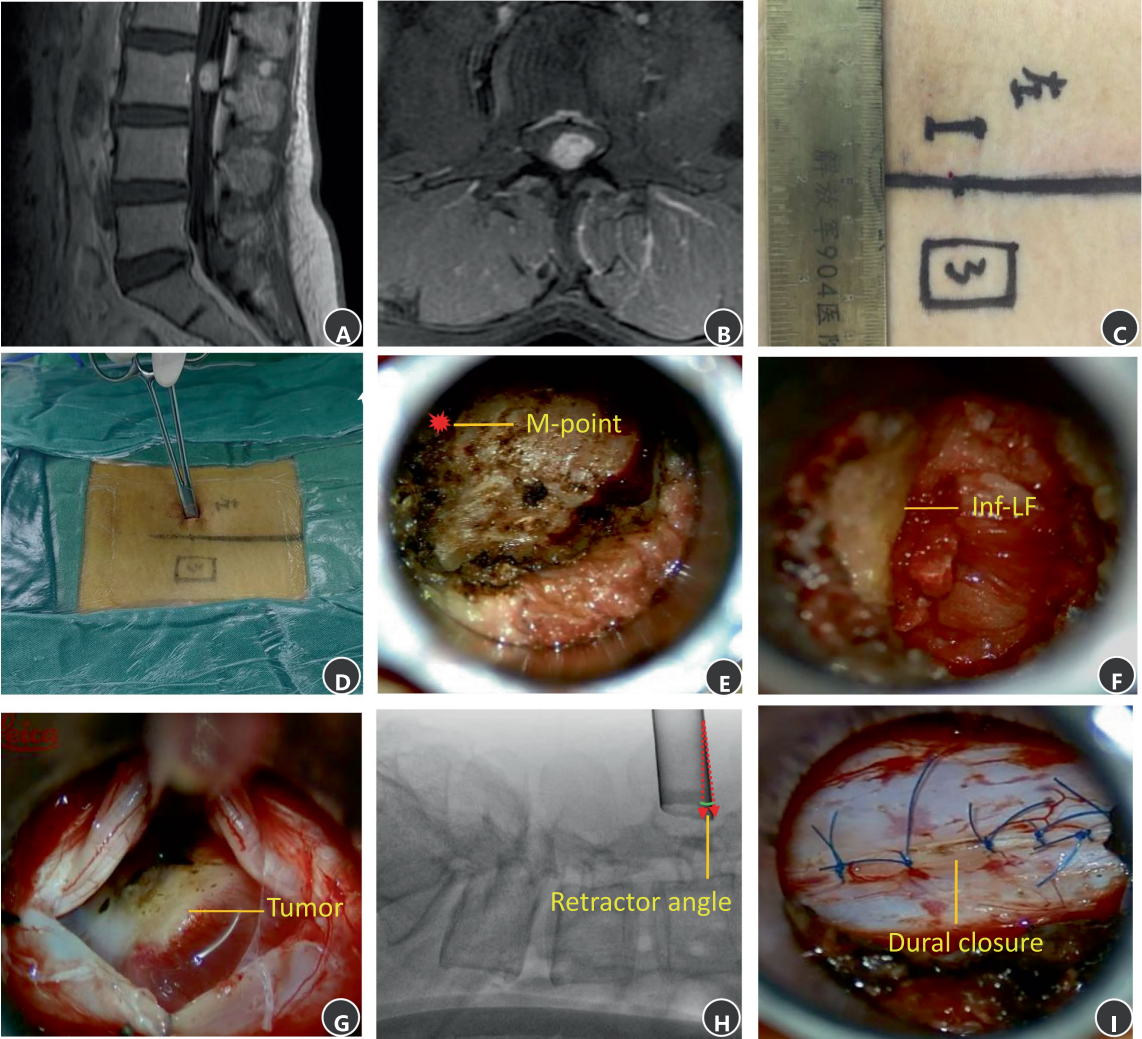
MRI and intraoperative surgical photos of schwannoma at the L3 level. **(A)** A sagittal T1 contrast image. **(B)** An axial T1 contrast image. **(C)** Surgical incision. **(D)** Palpating the M-point. **(E)** The M-point lies at the cephalad aspect of the operative field. **(F)** The inferior boundary of previous segment ligamentum flavum at the cephalad aspect of the operative field. **(G)** The main tumor mass is exposed and visualized at the center of the surgical corridor. **(H)** An intraoperative X-ray image showing retractor angle after exposed the main tumor mass. **(I)** Interrupted suture of the dura.

A 53-year-old female patient was diagnosed with an intraspinal schwannoma at L3 (Figures 5A,B) and underwent minimally invasive percutaneous resection (Case 14, Table 1). The incision was designed as shown in Figures 5C,D. Intraoperatively, the M-point at the superior margin of the L3 canal was identified cephalad (Figure 5E). After laminectomy, the inferior edge of the L2 ligamentum flavum was exposed (Figure 5F). The dura was opened, revealing the tumor centrally (Figure 5G). Retractor angulation was documented (Figure 5H). After the tumor was totally removed, the dura was sutured intermittently (Figure 5I).

## 4 Discussion

Advancements in minimally invasive techniques have established the microscopic tubular approach for resecting intraspinal benign tumors (6–9). However, variable tumor size and location, combined with the lack of a standard trajectory, make precise localization an essential consideration. The fixed tubular retractor (inner diameter: 1.6–1.8 cm) limits field-of-view and prevents full visualization of posterior spinal structures, increasing disorientation risk when only one structure is visible. In contrast, anatomical junctions where distinct structures converge provide critical and reliable spatial guidance (10–15).

Therefore, our technique utilizes the superior and inferior edges of the lamina as key bony landmarks. These landmarks guide surgical trajectory and correct incision deviation prior to dural exposure. The spatial relationship between tumor poles and ligamentum flavum boundaries (or S-point) then directs bone removal. This stepwise anatomic navigation ensures tumor centering upon dural opening. These landmarks proved reliable for localizing and exposing intraspinal schwannomas.

The M-point and S-point, defined as the junctions of the spinous process base with the superior and inferior lamina edges, respectively, are constant and easily palpable bony landmarks with minimal anatomical variation. They provide reliable intraoperative reference for initial incision placement and trajectory correction (10, 11). Preoperative MRI was used to select the optimal landmark (M-point or S-point) based on the tumor’s closest point (superior pole, inferior pole, or midpoint) to the lamina. Six theoretical spatial configurations between these points and the tumor guided landmark selection. Among the 17 cases, the M-point served as the cranial reference in 4 cases and the midpoint in 2 cases; the S-point was the midpoint in 7 cases and caudal reference in 3 cases. One case with tumor spanning both edges used the lamina midpoint. Of note, a minor offset (0–4 mm) from the ideal point was occasionally observed. Thus, final trajectory adjustment relied on surgical experience to account for this discrepancy.

Laminectomy was initiated from the preoperatively selected landmark (M-point or S-point), with the extent of bone removal planned based on preoperative MRI measurements between the tumor poles and the nearest boundary of the ligamentum flavum or S-point. The distance from tumor to ligamentum flavum ranged from 2.4 to 6.6 mm. In some cases, bone removal extended slightly beyond the ligamentum flavum under direct visual guidance using the drill tip as a reference. Surgeons were cautious to cease drilling once the predefined boundary was reached to avoid overexposure. This approach enabled precise laminectomy, followed by ligamentum flavum resection and dural opening. After centering the tumor, lateral fluoroscopy confirmed a mean retractor inclination angle of less than 10° in all cases, indicating high targeting accuracy. Compared to other intraoperative localization techniques, the method described here demonstrates distinct advantages. While navigation based on intraoperative computed tomography (iCT) registration can improve accuracy, it typically necessitates additional intraoperative fluoroscopic steps, potentially increasing radiation exposure (16). Microscope-based augmented reality (AR) assistance, although providing intuitive intraoperative projection, involves workflow integration steps such as image fusion and microscope calibration, which may prolong operative time (17). In contrast, the activated carbon marking technique combined with CT-guided fluoroscopy offers a preoperative localization method but lacks precision in defining the exact extent of bone resection required. Furthermore, it depends on an additional preoperative CT scan for marker placement (18).

All surgical procedures in this series were performed under direct visualization through a microscope-assisted tubular retractor system. The operations were conducted in an air medium, with tumor resection initiated after opening the dura and releasing a portion of cerebrospinal fluid. Unlike fully endoscopic lumbar surgery, which typically requires sustained irrigation, this technique did not necessitate continuous saline irrigation to maintain a clear visual field. In endoscopic procedures, the pressure from continuous irrigation has been associated with complications such as dural tears, outflow obstruction, and subsequent postoperative issues including cervical and headache pain, seizure episodes, as well as intracranial and retinal hemorrhage (19–21). Notably, none of the patients in our cohort experienced such complications.

It should be noted that this study exclusively included patients with intraspinal schwannomas and was limited to the lumbar region. However, benign intradural extramedullary tumors also exhibit a considerable incidence in the cervical and thoracic spine. Several reports have described minimally invasive approaches for cervical and thoracic lesions (22, 23). Future studies may therefore explore the application of the localization method presented here for tumors in these spinal regions. Although minimally invasive resection of spinal meningiomas has been described in the literature (24, 25), such cases were excluded from this series due to the frequent involvement of the dura mater and the associated complexity of dural reconstruction. Future research could assess the applicability of this technique to carefully selected meningiomas or to conditions such as thoracic ossified ligamentum flavum (26–29). Further validation under appropriate ethical approval will be necessary to evaluate its efficacy for these expanded indications.

This study has several limitations. First, preoperative identification of bony landmarks relied mainly on sagittal T1-weighted MRI, which offers suboptimal osseous visualization. Using transitional zones as proxies for laminar edges may introduce error; future studies could improve accuracy by integrating 3D CT-MRI fusion. Second, distance measurements were performed on spinous process-traversing MRI slices, but the non-uniform geometry of the ligamentum flavum and possible misalignment between tumor poles and landmarks could affect measurement reliability. Third, the small sample size and lack of a control group limit the generalizability of the favorable targeting accuracy observed. Large multicenter RCTs are needed for validation. Finally, the technique’s dependence on spatial relationships between bony and ligamentous landmarks may challenge less-experienced surgeons. Developing a zonal anatomical classification could help standardize approach selection and facilitate training.

## 5 Conclusion

This study demonstrates that the lamina and ligamentum flavum boundaries are reliable anatomical landmarks for precise localization and safe resection of lumbar intraspinal schwannomas using a minimally invasive tubular technique. This method resulted in favorable short-term clinical outcomes, high targeting accuracy, and minimal complications. However, further validation through larger cohorts and long-term studies is warranted to confirm its generalizability and durability.

## Data Availability

The original contributions presented in the study are included in the article/supplementary material, further inquiries can be directed to the corresponding authors.
